# Supplementary material for: Urinary tract colonization is enhanced by a plasmid that regulates uropathogenic Acinetobacter baumannii chromosomal genes
Source: Nat Commun. 2019 Jun 24;10:2763. doi: 10.1038/s41467-019-10706-y (PMC6591400; doi:10.1038/s41467-019-10706-y)
Supplement: Supplementary file 1 — Supplementary Information [file 41467_2019_10706_MOESM1_ESM.pdf]

Supplementary Information

Urinary tract colonization is enhanced by a plasmid that regulates uropathogenic *Acinetobacter baumannii* chromosomal genes

Di Venanzio et al.

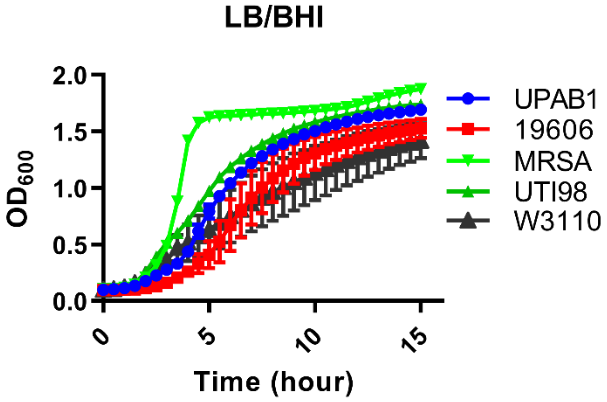

Supplementary Figure 1. UPAB1 and 19606 strains display indistinguishable growth in rich media. Growth curves of UPAB1, 19606, MRSA 1369, *E. coli* UTI89 and *E. coli* W3110 in rich media (LB or BHI) as measured by OD600. The number of independent data points represented is four. Data represent mean and standard deviation values. Source data are provided as a Source Data file.

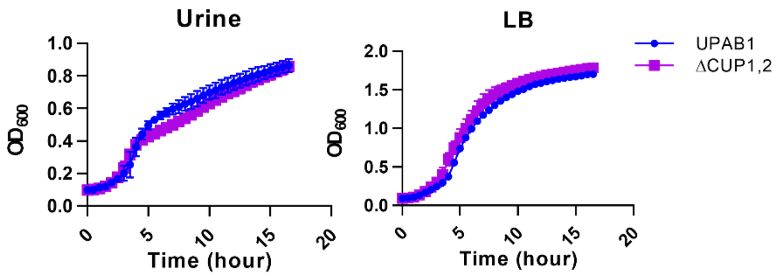

Supplementary Figure 2. UPAB1 and ΔCUP1,2 strain display identical growth in rich medium and urine. Growth curves of wild-type UPAB1 and ΔCUP1,2 mutant strain in healthy pooled urine (left panel) or rich media (LB, right panel) as measured by OD600. The number of independent data points represented is four. Data represent mean and standard deviation values. Source data are provided as a Source Data file.

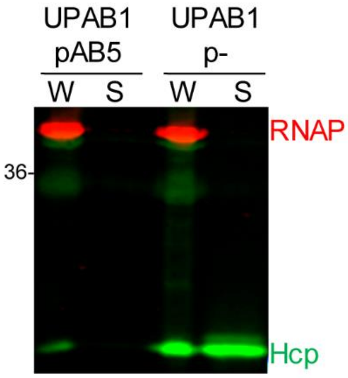

Supplementary Figure 3. pAB5 represses the T6SS in UPAB1. Western blot assays probing for Hcp (green) expression and secretion in whole-cell (W) or supernatants (S) of wild-type UPAB1 and UPAB1p- mutant strain. RNA polymerase (RNAP, red) was used as a loading and lysis control. Source data are provided as a Source Data file.

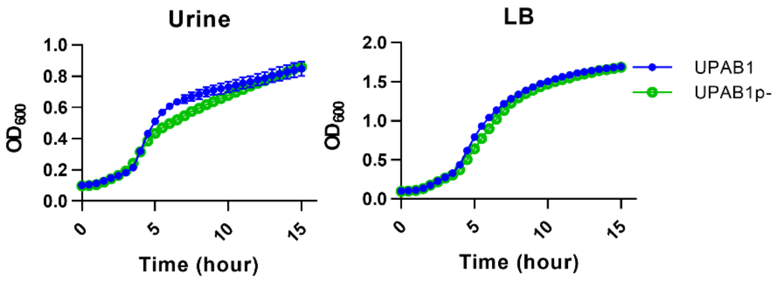

Supplementary Figure 4. UPAB1 and UPAB1p- strain display identical growth in rich medium and urine. Growth curves of wild-type UPAB1 and UPAB1p- mutant strain in healthy pooled urine (left panel) or rich media (LB, right panel) as measured by OD600. The number of independent data points represented is four. Data represent mean and standard deviation values. Source data are provided as a Source Data file.

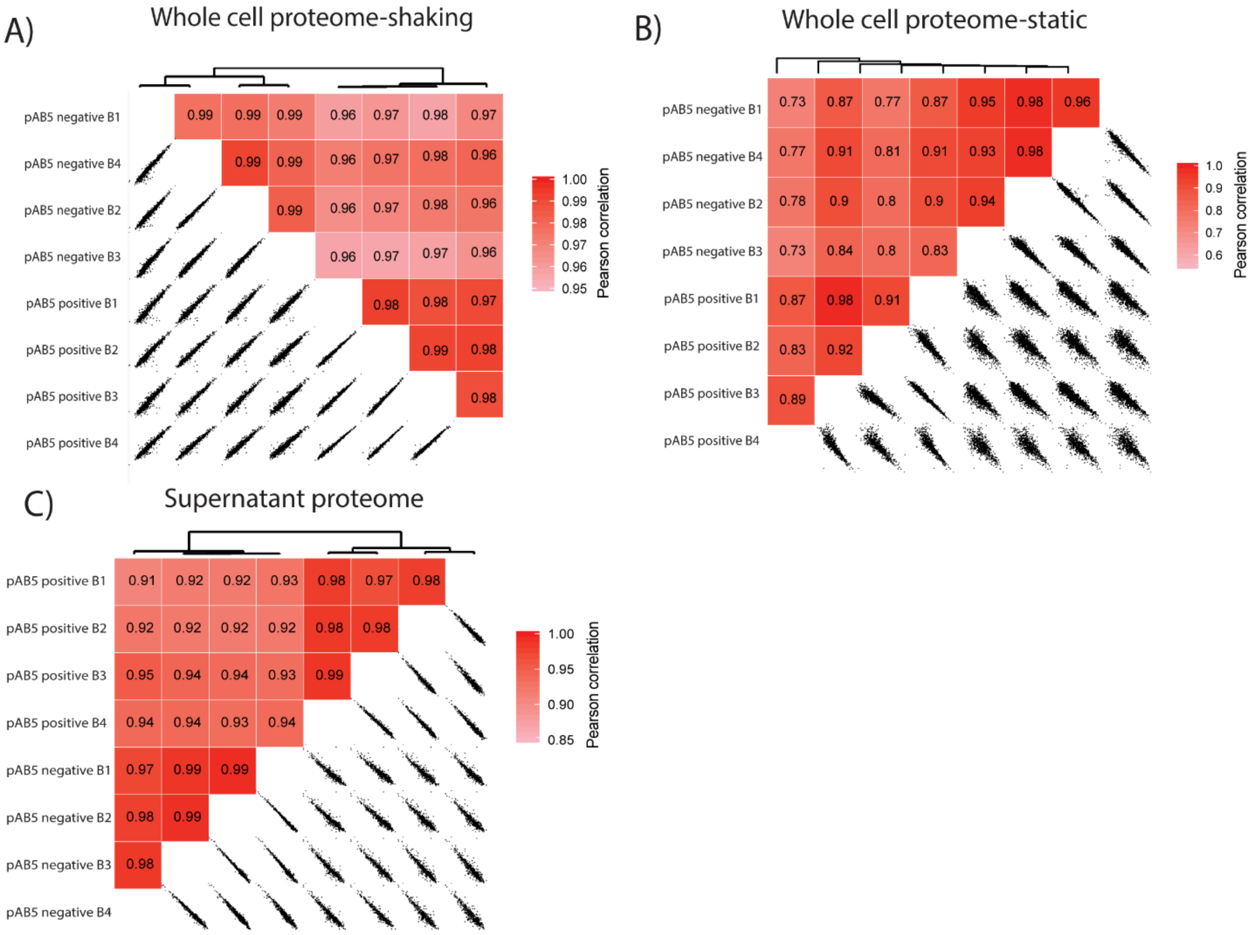

Supplementary Figure 5. Pearson correlation analysis of proteomics biological replicates in the absent/presence of pAB5. To assess the reproducibility of the proteome samples, heat maps and correlation plots are provided for; A) Whole proteome replicates grown under shaking demonstrate a mean correlation between biological replicate of 0.96 and a correlation of 0.99 within biological groups. B) Whole proteome replicates grown under static condition demonstrate a mean correlation between biological replicate of 0.86 and a correlation of 0.91 within biological groups. C) Secretome replicates demonstrated a mean correlation of 0.92 and a correlation of 0.98 within biological groups.

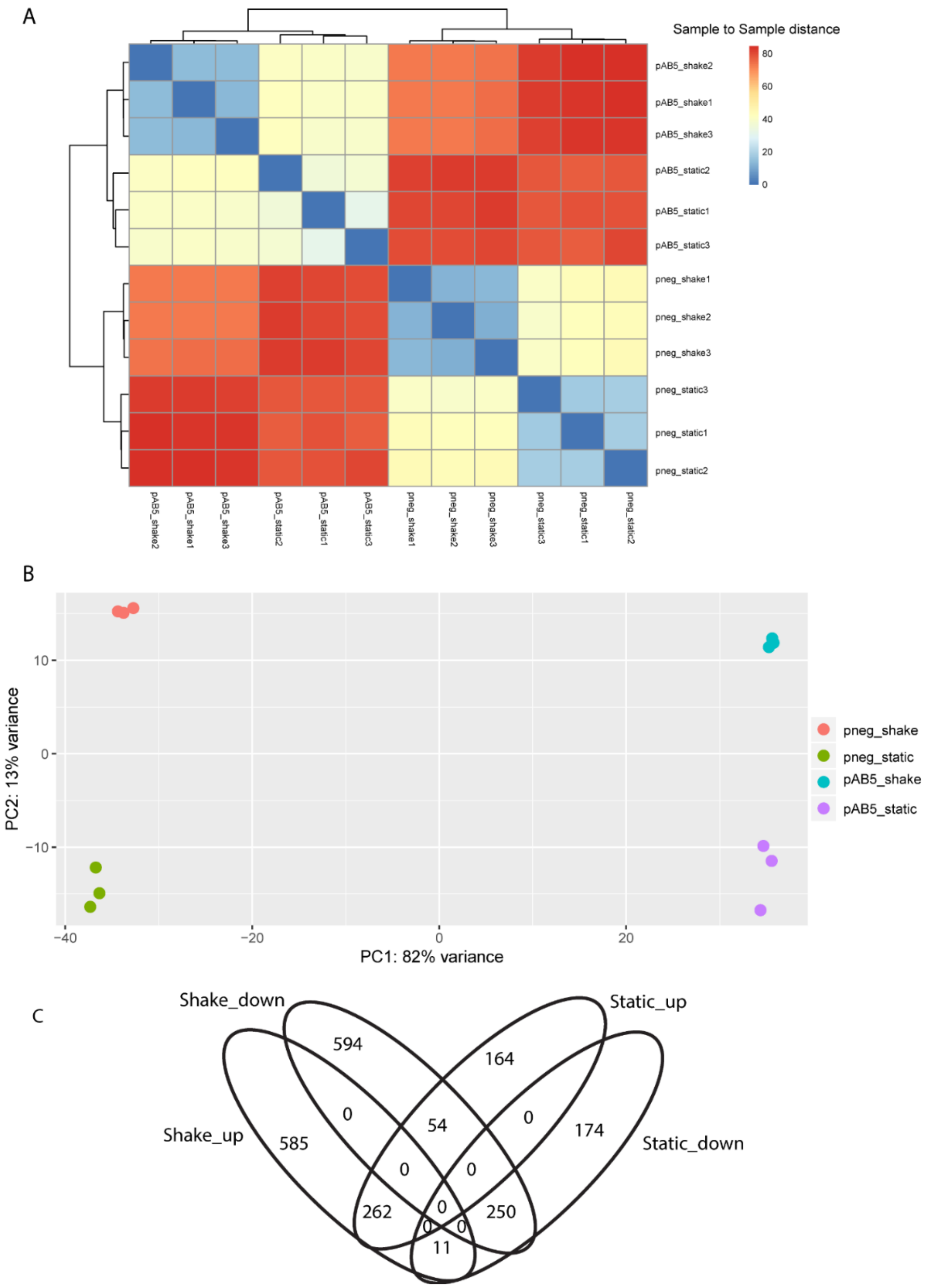

Supplementary Figure 6. Between group variation is much greater than between sample variation. Heatmap (A) and principal component analysis (B) of the variance stabilized transformed read counts used as input for DESeq2 analysis indicates that biological replicates cluster next to one another and that the presence of pAB5 and growth conditions drastically change the transcription profile of UPAB1. Venn-diagram (C) analysis of the DEGs with adjusted p-values <.1 indicates that there is some overlap between the transcriptional profile of UPAB1 in the various environments.

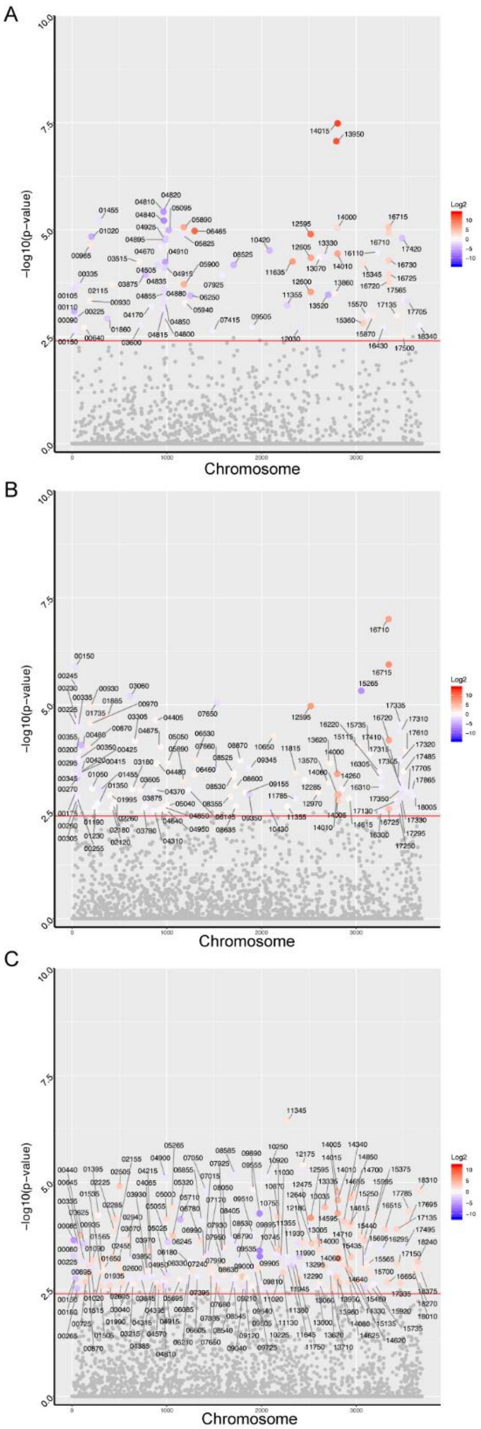

Supplementary Figure 7. pAB5 modulates several bacterial proteins. Quantitative proteome analysis of the effect of pAB5 on the secretome (A), the whole proteome with shaking (B) and whole proteome under static conditions (C). Manhattan plots demonstrating the significance of protein alteration, –log10(p-value), vs position in the genome are shown. The direction of the protein alteration is colored coded according the provided heat map. Full data are shown.

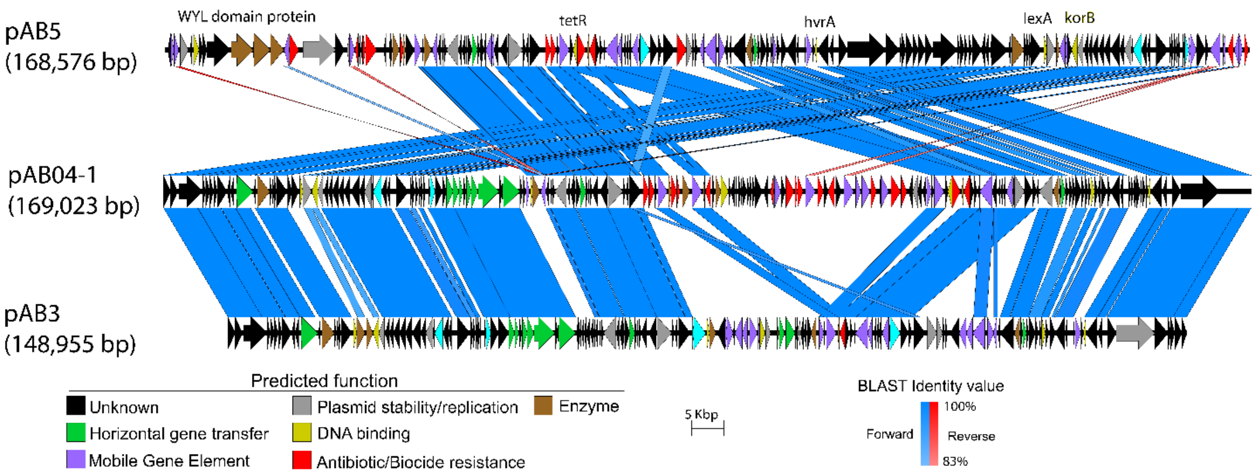

Supplementary Figure 8. *A. baumannii* LCPs share common structural features. Assembled plasmids from PacBio sequencing of UPAB1 (pAB5), Ab04 (pAB04-1) and Ab17978 (pAB3) strains. The transcriptional regulators identified in pAB5 are listed in the top of the figure.

Supplementary Table 1. Summary of 13 *Ab* epidemiological studies

				Number of isolate per anatomical site (n)					
	Study Dates	Location	Ref	Urinary	Respiratory	SST/MSK	Endovascular	Other	**Total**
Siau, et al, 1996	1/1990-11/1994	Hong Kong	1	1434	2724	1856	436	596	**7046**
Ruiz, et al, 1999	1991-1996	Spain	2	439	434	325	195	139	**1532**
Iregbu, et al, 2002	2001	Nigeria	3	17	0	37	4	0	**58**
Tognim, et al, 2004	1/1997-12/2001	Latin America	4	74	299	35	380	38	**826**
Perencevich, et al, 2008	1/1998-12/2005	USA	5	260	505	260	361	58	**1444**
McCracken, et al, 2011	2007-2009	Canada	6	4	22	5	35	0	**66**
Sinha, et al, 2013	8/2010-7/2011	India	7	19	0	52	32	37	**140**
Munoz-Price, et al, 2013	1/1994-12/2011	USA	8	444	2194	622	819	405	**4484**
Al Mobarak, et al, 2014	1/2010-12/2013	Saudi Arabia	9	127	315	497	110	127	**1176**
Fallah, et al, 2017	5/2015-7/2016	Iran	10	13	59	15	12	1	**100**
Biglari, et al, 2017	10/2010-4/2011	Malaysia	11	17	54	74	10	12	**167**
Matsui, et al, 2018	10/2012-3/2013	Japan	12	57	498	34	13	43	**645**
Current BJC Study	1/2007-7/2017	USA		505	771	726	237	34	**2273**
**Pooled Total**	**--**	**--**		**3410**	**7875**	**4538**	**2644**	**1490**	**19957**
**% Pooled Total**	**--**	**--**		**17.1%**	**39.5%**	**22.7%**	**13.2%**	**7.5%**	**100%**

Supplementary Table 2. Secreted proteins differentially regulated by pAB5.

D1G37	Name	Function	Signal	Fold change
Up-regulated proteins				
110	Cell surface protein Ata	T5SS	NO	4.59
1455	OmpW family protein		YES	1.64
6250	M23 family metallopeptidase		YES	4.02
17420	Type I 3-dehydroquinate dehydratase CDS		YES	3.63
19690	Hypothetical protein		YES	9.42
Down-regulated proteins				
965	Hemagglutinin	T5SS	NO	1.55
2115	Insulinase family protein		YES	0.98
4670	DUF3108 domain-containing protein		YES	0.97
5890	Hypothetical protein	T6SS	YES	6.00
5900	Rhs element Vgr	T6SS	NO	5.56
6465	LysM peptidoglycan-binding domain-containing protein	T6SS	NO	10.46
11635	PAAR domain-containing protein	T6SS	NO	7.09
12595	Curli production assembly transport component CsgG		YES	9.91
12600	Probable lipoprotein		YES	6.80
12605	Phosphonate ABC transporter phosphate-binding periplasmic component		YES	6.32
13860	Filamentous hemagglutinin N-terminal domain-containing protein	T5SS	NO	1.08
13950	VgrG	T6SS	NO	11.63
14000	Hypothetical protein	T6SS	YES	2.52
14010	TssC	T6SS	NO	6.45
14015	Hcp	T6SS	NO	12.26
15345	PrpD	CUP1	YES	1.93
15360	PrpA	CUP1	YES	3.27
16110	TonB-dependent siderophore receptor CDS		YES	0.92
16710	CupD	CUP2	YES	3.08
16715	CupC	CUP2	NO	3.91
16720	CupB	CUP2	YES	2.51
16725	CupA	CUP2	YES	2.76
16730	FimF	CUP2	YES	3.43

Supplementary Table 3. Proteins differentially regulated by pAB5 on SHc.

D1G37	Name	Function	Fold change
Up-regulated proteins			
270	TsaE		3.26
480	Hypothetical protein		4.10
1455	OmpW family protein		1.75
3060	Hypothetical protein		2.33
9350	Hypothetical protein		2.69
15265	Amino acid ABC transporter ATP-binding protein		6.52
Down-regulated proteins			
150	Membrane alanine aminopeptidase N		1.62
175	5-carboxymethyl-2-hydroxymuconate Delta-isomerase		1.28
200	Aldehyde dehydrogenase family protein		1.60
225	Peptidyl-prolyl cis-trans isomerase		1.26
230	UDP-2,3-diacylglucosamine diphosphatase		1.09
245	Oxygen-insensitive NAD(P)H nitroreductase		1.02
295	Hypothetical protein		0.40
335	Bifunctional aconitate hydratase 2/2-methylisocitrate dehydratase		1.06
415	Ubiquinone biosynthesis monooxygenase UbiB		1.19
425	Flavin reductase		1.70
870	NirD/YgiW/YdeI family stress tolerance protein		1.34
970	ShlB/FhaC/HecB family hemolysin secretion/activation protein CDS	T5SS	1.03
1735	TonB-dependent siderophore receptor		2.43
1885	LysR family transcriptional regulator		1.09
5890	Hypothetical protein		1.48
6530	HutG		1.50
7650	D-amino acid dehydrogenase small subunit		1.82
10430	Type IV fimbrial biogenesis protein PilY1		1.48
10650	N-acetyl-L,L-diaminopimelate deacetylase		1.15
12595	Curli production assembly transport component CsgG		6.93
11815	PgaB	PNAG	1.16
14000	Hypothetical protein	T6SS	6.48
14005	TssB	T6SS	3.49
14010	TssC	T6SS	4.90
14060	ClpV	T6SS	5.18
16710	CupD	CUP2	6.53
16715	CupC	CUP2	7.96
16720	CupB	CUP2	6.44
16725	CupA	CUP2	4.30
17350	Outer membrane porin, OprD family		1.89

Supplementary Table 4. Genes differently regulated by pAB5 in STc.

D1G37	Fold Change	Name	Function
09890	5.70	hypothetical protein	
15260	5.47	amino acid ABC transporter permease	
09895	5.31	molecular chaperone DnaJ	
09900	5.05	hypothetical protein	
09905	5.04	hypothetical protein	
09910	5.03	hypothetical protein	
09885	4.39	hypothetical protein	
15265	4.04	amino acid ABC transporter ATP-binding protein	
00085	3.23	cell envelope biogenesis protein OmpA	
12500	3.23	hypothetical protein	
13875	3.17	TetR/AcrR family transcriptional regulator	
00255	3.06	RNA chaperone Hfq	
09915	3.00	hypothetical protein	
00080	2.98	T1SS secreted agglutinin RTX	
00185	2.93	iron-containing alcohol dehydrogenase	
13870	2.67	hypothetical protein	
14065	**-4.60**	TssA	T6SS
14075	**-4.39**	tssL	T6SS
14070	**-4.22**	TssK	T6SS
14025	**-4.18**	TssF	T6SS
14030	**-4.12**	TssG	T6SS
14020	**-4.07**	TssE	T6SS
14035	**-3.98**	hypothetical protein	T6SS
14015	**-3.92**	Hcp	T6SS
14060	**-3.82**	ClpV	T6SS
14010	**-3.81**	TssC	T6SS
14080	**-3.68**	hypothetical protein	T6SS
14040	**-3.67**	TssM	T6SS
13955	**-3.59**	hypothetical protein	T6SS
14045	**-3.50**	TagF	T6SS
14050	**-3.47**	tagN	T6SS
14085	**-3.45**	tagX	T6SS
00935	**-3.33**	TetR/AcrR family transcriptional regulator	
14005	**-3.33**	tssB	T6SS
14000	**-3.32**	hypothetical protein	
12600	**-3.27**	Probable lipoprotein	
13960	**-3.10**	hypothetical protein	T6SS
15360	**-1.79**	prpA	CUP1
15345	**-1.62**	prpD	CUP1

Supplementary Table 5. Strain list

Strain	Description	Ref
UPAB1	MDR Urine isolate with pAB5 plasmid	This study
UPAB1p-	Derivative strain without pAB5	This study
UPAB1pAB3	Transconjugant strain	This study
UPAB1pAB4	Transconjugant strain	This study
UPAB1ΔCUP1,2	Unmarked deletion of *ABCD* genes from CUP1 and CUP2 locus	This study
MRSA 1369	Urine isolate	[13]
E. faecalis O671RF	Urine isolate	[13]
E. coli UTI89	Urine isolate	Hultgren lab

Supplementary Table 6. Primer list

CUP1 DS F FRT	ATAATGTGTATGATCATTTGACTTTTTTAGTATTAATTAAAAATTATTCATTCTATAAAGCCTACTACTTACTACTAAGATCTAGTGTTTAGAAGAAATA
CUP1 US R FRT	AAAATAATTTCATGATGTTTTACCATATGGTATAAATTAAAATAAAAACCGCATTTATCAATAAAAAAATAACCAATCAAAATACATAAAAATATAATGTT
CUP1out F	GCAAATTGTGATCTATTTCCC
CUP1 out R	CCAAACTCCAGATGATCTTTTATTG
CUP2 US R Frt	TTTGTTTAAAAAGTGTTAGCATTGTTACATTACTTTTCAAGATGTCAAACACTGGAAAAGTAAGGAATTAAACTTTTATCCAGACTTTTGAGAAAACACT
CUP2 DS F frt	ATATTCCTATCGAGTTAAGTATGAAATTTTTAGAAAGTAAAAGTTATTATTAATATTTAATTATTTGAAAATTATTAATTTTATTAACTATTGAATATGA
CUP2 out F	ATGTGACTTTTTGGCTAGCC
CUP2 out R	TCTTATCTCTATGATTCCTTTCTTC

Supplementary References

1. Siau, H., Yuen, K. Y., Wong, S. S., Ho, P. L. & Luk, W. K. The epidemiology of acinetobacter infections in Hong Kong. *J. Med. Microbiol.* **44,** 340–7 (1996).

2. Ruiz, J. *et al.* Evolution of resistance among clinical isolates of Acinetobacter over a 6-year period. *Eur. J. Clin. Microbiol. Infect. Dis.* **18,** 292–5 (1999).

3. Iregbu, K. C., Ogunsola, F. T. & Odugbemi, T. O. Infections caused by Acinetobacter species and their susceptibility to 14 antibiotics in Lagos University Teaching Hospital, Lagos. *West Afr. J. Med.* **21,** 226–9 (2002).

4. Tognim, M. C. B. *et al.* Resistance trends of Acinetobacter spp. in Latin America and characterization of international dissemination of multi-drug resistant strains: five-year report of the SENTRY Antimicrobial Surveillance Program. *Int. J. Infect. Dis.* **8,** 284–291 (2004).

5. Perencevich, E. N. *et al.* Summer Peaks in the Incidences of Gram-Negative Bacterial Infection Among Hospitalized Patients. *Infect. Control Hosp. Epidemiol.* **29,** 1124–31 (2008).

6. McCracken, M. *et al.* Characterization of Acinetobacter baumannii and meropenem-resistant Pseudomonas aeruginosa in Canada: results of the CANWARD 2007–2009 study. *Diagn. Microbiol. Infect. Dis.* **69,** 335–341 (2011).

7. Sinha, N., Agarwal, J., Srivastava, S. & Singh, M. Analysis of carbapenem-resistant Acinetobacter from a tertiary care setting in North India. *Indian J. Med. Microbiol.* **31,** 60–3 (2013).

8. Munoz-Price, L. S. *et al.* Eighteen Years of Experience With Acinetobacter baumannii in a Tertiary Care Hospital*. *Crit. Care Med.* **41,** 2733–2742 (2013).

9. Al Mobarak, M. F. *et al. Antimicrobial Resistance Patterns Among Acinetobacter baumannii Isolated From King Abdulaziz Hospital, Jeddah,Saudi Arabia, Four-Year Surveillance Study (2010-2013)*. *Egyptian Journal of Medical Microbiology* **23,** (2014).

10. Fallah, A. *et al.* Frequency of bap and cpaA virulence genes in drug resistant clinical isolates of Acinetobacter baumannii and their role in biofilm formation. *Iran. J. Basic Med. Sci.* **20,** :849-855 (2017).

11. Biglari, S. *et al.* Antimicrobial Resistance Mechanisms and Genetic Diversity of Multidrug-Resistant *Acinetobacter baumannii* Isolated from a Teaching Hospital in Malaysia. *Microb. Drug Resist.* **23,** 545–555 (2017).

12. Matsui, M. *et al.* Distribution and Molecular Characterization of Acinetobacter baumannii International Clone II Lineage in Japan. *Antimicrob. Agents Chemother.* **62,** (2018).

13. Flores-Mireles, A. L. *et al.* Fibrinogen Release and Deposition on Urinary Catheters Placed during Urological Procedures. *J. Urol.* **196,** 416–421 (2016).
