# Supplementary material for: Urinary tract colonization is enhanced by a plasmid that regulates uropathogenic Acinetobacter baumannii chromosomal genes
Source: Nat Commun. 2019 Jun 24;10:2763. doi: 10.1038/s41467-019-10706-y (PMC6591400; doi:10.1038/s41467-019-10706-y)
Supplement: Supplementary file 3 — Description of Additional Supplementary Files [file 41467_2019_10706_MOESM3_ESM.pdf]

Description of Additional Supplementary Files

File Name: Supplementary Data 1
Description: **Proteins differentially regulated by pAB5 on STc.** Quantitative proteomic analysis of *A. baumannii* UPAB1 whole cell proteome with and without pAB5 under static growth. A list of the selected proteins and accession numbers shown in figure 6C is provided.

File Name: Supplementary Data 2
Description: **Genes differently regulated by pAB5 in SHc.** Transcriptomic analysis of *A. baumannii* UPAB1 whole cell proteome with and without pAB5 under shaking growth. A list of the selected genes and accession numbers shown in figure 7A is provided.

File Name: Supplementary Data 3
Description: LFQ based analysis of *A. baumannii* UPAB1 Whole cell proteome with and without pAB5 under shaking growth. A total of the 1638 protein groups were observed across eight biological replicates, four with pAB5 and four without pAB5. LFQ experiment 1 to 4 correspond to UPAB1 without plasmid biological replicate 1 to 4 and LFQ experiment 5 to 8 correspond to UPAB1 with pAB5 biological replicate 1 to 4. For each protein group the LFQ values, t-test significance, number of peptides, score, number of MS/MS events, intensity values, identification type and protein name gene identified using Maxquant are provided. The fold change difference in the mean log2(LFQ) value of biological conditions is provided as the difference between condition while the t-test p-value is presented as the -log10 value.

File Name: Supplementary Data 4
Description: LFQ based analysis of *A. baumannii* UPAB1 Secretome with and without pAB5. A total of the 908 protein groups were observed across eight biological replicates, four with pAB5 and four without pAB5. LFQ experiment 1 to 4 correspond to the secretome of UPAB1 without plasmid biological replicate 1 to 4 and LFQ experiment 5 to 8 correspond to secretome of UPAB1 with pAB5 biological replicate 1 to 4. For each protein group the LFQ values, t-test significance, number of peptides, score, number of MS/MS events, intensity values, identification type and protein name gene identified using Maxquant are provided. The fold change difference in the mean log2(LFQ) value of biological conditions is provided as the difference between condition while the t-test p-value is presented as the -log10 value.

File Name: Supplementary Data 5
Description: LFQ based analysis of *A. baumannii* UPAB1 Whole cell proteome with and without pAB5 under static growth. A total of the 1903 protein groups were observed across eight biological replicates, four with pAB5 and four without pAB5. LFQ experiment 1 to 4 correspond to UPAB1 without plasmid biological replicate 1 to 4 and LFQ experiment 5 to 8 correspond to UPAB1 with pAB5 biological replicate 1 to 4. For each protein group the LFQ values, t-test significance, number of peptides, score, number of MS/MS events, intensity values, identification type and protein name gene identified using Maxquant are provided. The fold change difference in the mean log2(LFQ) value of biological conditions is provided as the difference between condition while the t-test p-value is presented as the -log10 value.
