# Supplementary material for: Urinary tract colonization is enhanced by a plasmid that regulates uropathogenic Acinetobacter baumannii chromosomal genes
Source: Nat Commun. 2019 Jun 24;10:2763. doi: 10.1038/s41467-019-10706-y (PMC6591400; doi:10.1038/s41467-019-10706-y)
Supplement: Supplementary file 9 — Reporting Summary [file 41467_2019_10706_MOESM9_ESM.pdf]

nature research

Corresponding author(s): Mario F. Feldman

Last updated by author(s): May 22, 2019

Reporting Summary

Nature Research wishes to improve the reproducibility of the work that we publish. This form provides structure for consistency and transparency in reporting. For further information on Nature Research policies, see Authors & Referees and the Editorial Policy Checklist.

Statistics

For all statistical analyses, confirm that the following items are present in the figure legend, table legend, main text, or Methods section.

n/a	Confirmed	
☐	☒	The exact sample size (*n*) for each experimental group/condition, given as a discrete number and unit of measurement
☐	☒	A statement on whether measurements were taken from distinct samples or whether the same sample was measured repeatedly
☐	☒	The statistical test(s) used AND whether they are one- or two-sided<br>*Only common tests should be described solely by name; describe more complex techniques in the Methods section.*
☒	☐	A description of all covariates tested
☐	☒	A description of any assumptions or corrections, such as tests of normality and adjustment for multiple comparisons
☐	☒	A full description of the statistical parameters including central tendency (e.g. means) or other basic estimates (e.g. regression coefficient) AND variation (e.g. standard deviation) or associated estimates of uncertainty (e.g. confidence intervals)
☒	☐	For null hypothesis testing, the test statistic (e.g. *F*, *t*, *r*) with confidence intervals, effect sizes, degrees of freedom and *P* value noted<br>*Give P values as exact values whenever suitable.*
☒	☐	For Bayesian analysis, information on the choice of priors and Markov chain Monte Carlo settings
☒	☐	For hierarchical and complex designs, identification of the appropriate level for tests and full reporting of outcomes
☒	☐	Estimates of effect sizes (e.g. Cohen's *d*, Pearson's *r*), indicating how they were calculated

Our web collection on statistics for biologists contains articles on many of the points above.

Software and code

Policy information about availability of computer code

Data collection

No software was used.

Data analysis

Sequencing was performed at the McGill University and Genome Quebec Innovation Center. Genome annotation was conducted using the NCBI Prokaryotic Genome Annotation Pipeline.
Proteomic data analysis. MS data were processed using MaxQuant (v1.5.3.30). The resulting outputs were processed within the Perseus (v1.5.0.9)41 analysis environment to remove reverse matches and common proteins contaminates prior to further analysis. For LFQ comparisons missing values were imputed with a downshift of 2.5 and width of 0.3 standard derivations. Statistically assessment of alterations between conditions was done using two sample t-test within Perseus with a Benjamini Hochberg correction FDR of 0.05. Pearson correlations and Perseus outputs were visualized using R (https://www.r-project.org/).
Transcriptomic analysis. Pooled cDNA libraries were submitted to the Center for Genome Sciences & Systems Biology at Washington University in St. Louis School of Medicine. Samples were sequenced on an Illumina NextSeq 550 system to obtain 1x75 bp sequences. Raw reads were demultiplexed by barcodes and had adapters removed with trimmomatic v.38using the command “java -Xms1024m -Xmx1024m -jar <trimmomatic_jar> SE -phred33 -trimlog <trimlog_output> <multiplexed_read> <trimmed_read> ILLUMINACLIP:/opt/apps/trimmomatic/0.36/adapters/NexteraPE-PE.fa:2:30:10 LEADING:3 TRAILING:3 SLIDINGWINDOW:4:15 MINLEN:36”45. The UPAB1 genome (Accession CP032215-20) was converted into a bowtie2 v.2.3.4.1 index with command “bowtie2-build UPAB1.fasta <index>” and the trimmed reads were aligned to it with command “bowtie2 -x <index> -U <trimmed_read> -S <sam_output> 2> <bowtie2_log>” to generate SAM files46. Count matrices were generated using FeatureCounts within subread v1.5.3 with command “srun featureCounts -a <SAF_file> -F SAF -o <count_output> <sam_file>”47. Differential expression analysis of the count matrix was performed using DESeq248. Per the DESeq2 vignette (http://bioconductor.org/packages/devel/bioc/vignettes/DESeq2/inst/doc/DESeq2.html), genes with counts <10 were discarded from differential expression analysis. Variance stabilizing transformation of read count was analyzed as a heatmap and principal component figure. DEG analysis was performed by comparing UPAB1 with pAB5 versus UPAB1 cured of pAB5 separately for shaking and static growth. Per the DESeq2 vignette, genes with adjusted p-values <.1 were determined to be significantly differentially expressed. All statistical analyses were performed using GraphPad Prism (GraphPad Software Inc., La Jolla, CA).

For manuscripts utilizing custom algorithms or software that are central to the research but not yet described in published literature, software must be made available to editors/reviewers. We strongly encourage code deposition in a community repository (e.g. GitHub). See the Nature Research guidelines for submitting code & software for further information.

Data

Policy information about availability of data

All manuscripts must include a data availability statement. This statement should provide the following information, where applicable:

- Accession codes, unique identifiers, or web links for publicly available datasets
- A list of figures that have associated raw data
- A description of any restrictions on data availability

The mass spectrometry proteomics data have been deposited to the ProteomeXchange Consortium via the PRIDE42 partner repository with the dataset identifier PXD011302 and PXD011341. The whole-genome sequence project was deposited in the Sequence Read Archive (SRA) at the National Center for Biotechnology Information (NCBI) under the accession number PRJNA487603, and the whole-genome sequences were deposited in the GenBank database under the accession numbers CP032215-CP032220. Processed RNA-seq reads have been submitted to the Short Read Archive under BioProject PRJNA499107.

Field-specific reporting

Please select the one below that is the best fit for your research. If you are not sure, read the appropriate sections before making your selection.

☒ Life sciences ☐ Behavioural & social sciences ☐ Ecological, evolutionary & environmental sciences

For a reference copy of the document with all sections, see nature.com/documents/nr-reporting-summary-flat.pdf

Life sciences study design

All studies must disclose on these points even when the disclosure is negative.

Sample size	Mouse CAUTI experiments were conducted in 2 independent replicates of 5 mice per strain per time point per condition on different days. Ten mice are sufficient to detect statistically significant differences in bacterial burden. Mouse pneumonia experiments were conducted in 2 independent replicates of 10 mice per strain per time point per condition on different days.
Data exclusions	No data were excluded from the analysis.
Replication	All attemps of replication were successfull.
Randomization	We solely used female C57BL/6Crl mice for CAUTI experiments, as occlusion of the urethra by the prostate makes transurethral implant insertion and inoculation impossible in male mice. C57Bl/6Crl mice also retain the implant at a higher frequency than mice from other strains
Blinding	Blinding was not relevant to this study

Reporting for specific materials, systems and methods

We require information from authors about some types of materials, experimental systems and methods used in many studies. Here, indicate whether each material, system or method listed is relevant to your study. If you are not sure if a list item applies to your research, read the appropriate section before selecting a response.

Materials & experimental systems

n/a	Involved in the study	
☐	☒	Antibodies
☒	☐	Eukaryotic cell lines
☒	☐	Palaeontology
☐	☒	Animals and other organisms
☐	☒	Human research participants
☒	☐	Clinical data

Methods

n/a	Involved in the study	
☒	☐	ChIP-seq
☒	☐	Flow cytometry
☒	☐	MRI-based neuroimaging

Antibodies

Antibodies used	donkey anti-goat IRDye 800CW, donkey anti-rabbit IRDye 680LT, IRDye 680 RD Goat anti-mouse 926-6870 and IRDye 800CW Goat anr=ti-rabbit 926-32211 (LI-COR Biosciences). Mouse anti-E. coli RNA polymerase (Biolegend). anti HCP antibody was previously described (Weber et al, PlosOne, 2013). Rabbit anti-UPAB1 primary antibody (Antibody Research Corporation)
Validation	Anti-UPAB1 primary antibody was validated by End point ELISA by Antibody Research Corporation. A wester blot validation was performed by GDV.

Animals and other organisms

Policy information about studies involving animals; ARRIVE guidelines recommended for reporting animal research

Laboratory animals	Murine Model of A. baumannii Catheter-Associated UTI. Six- to 8-wk-old female C57BL/6 mice were obtained from Charles River Laboratories. Murine model of A. baumannii acute pneumonia. 9-week-old male C57BL/6 mice (Jackson Laboratories)
Wild animals	The study did not involve wild animals
Field-collected samples	The study did not involve samples collected from the field
Ethics oversight	All acute pneumonia experiments were approved by the Vanderbilt University Institutional Animal Care and Use Committee. All CAUTI studies were performed in accordance with the guidelines of the Committee for Animal Studies at Washington University School of Medicine.

Note that full information on the approval of the study protocol must also be provided in the manuscript.

Human research participants

Policy information about studies involving human research participants

Population characteristics	All clinical data was obtained through the Barnes Jewish Healthcare System medical database and chart review. As outlined in the methods, all cases in which Acinetobacter was reported in clinical cultures in our healthcare system from 1/2007-8/2017, were eligible for inclusion in our study. There were no exclusion criteria. Given the retrospective, observational nature of this study, it was exempt from the need to obtain consent.
Recruitment	No recruitment was performed in this retrospective analysis of clinical data.
Ethics oversight	Internal Review Board at Washington University School of Medicine in St Louis (IRB# 201707046)

Note that full information on the approval of the study protocol must also be provided in the manuscript.
